# Distracted by Previous Experience: Integrating Selection History, Current Task Demands and Saliency in an Algorithmic Model

**DOI:** 10.1007/s42113-024-00197-6

**Published:** 2024-02-29

**Authors:** Neda Meibodi, Hossein Abbasi, Anna Schubö, Dominik Endres

**Affiliations:** 1 Department of Psychology, Philipps-Universität Marburg, Gutenbergstraße 18, Marburg, 35037 Hessen Germany; 2 Biological Psychology and Neuropsychology, Universität Hamburg, Von-Melle-Park 11, Hamburg, 20146 Hamburg Germany

**Keywords:** Visual attention modeling, Selection history, Feature integrated theory, Integrated priority map, Self-information maximization, Ex-Gaussian distribution

## Abstract

Attention can be biased by previous learning and experience. We present an algorithmic-level model of this selection history bias in visual attention that predicts quantitatively how stimulus-driven processes, goal-driven control and selection history compete to control attention. In the model, the output of saliency maps as stimulus-driven guidance interacts with a history map that encodes learning effects and a goal-driven task control to prioritize visual features. The model works on coded features rather than image pixels which is common in many traditional saliency models. We test the model on a reaction time (RT) data from a psychophysical experiment. The model accurately predicts parameters of reaction time distributions from an integrated priority map that is comprised of an optimal, weighted combination of separate maps. Analysis of the weights confirms selection history effects on attention guidance. The model is able to capture individual differences between participants’ RTs and response probabilities per group. Moreover, we demonstrate that a model with a reduced set of maps performs worse, indicating that integrating history, saliency and task information are required for a quantitative description of human attention. Besides, we show that adding intertrial effect to the model (as another lingering bias) improves the model’s predictive performance.

## Introduction

Selective visual attention is a brain function that filters irrelevant sensory inputs to facilitate focusing on relevant items. Stimulus-driven and goal-driven mechanisms have traditionally been proposed to control the process of attention guidance. Object saliency and environment features shape the attentional process in a stimulus-driven manner while the goal-driven process is mostly controlled by observer intentions and preferences. In addition to goal-driven and stimulus-driven contributions also “selection history” can play a significant role in guiding attention toward a specific target (Theeuwes, 2019). Selection history (as a third mechanism of attentional guidance) comes into play when an object is emphasized just because of previous attendance in the same context (Awh et al., 2012). To clarify the distinction between goal-driven guidance and selection history, Theeuwes argued that selection history is a fast, effortless, and automatic version of attention control while goal-driven selection is slow and effortful (Theeuwes, 2018). The term “selection history” includes several phenomena that can neither be considered as goal-driven nor as stimulus-driven control, such as lingering effects, statistical learning, emotional and also reward-based biases (Failing & Theeuwes, 2018).

One special form of selection history has been investigated in Feldmann-Wüstefeld et al. (2015), Kadel et al. (2017), and Henare et al. (2020). These studies combined an associative learning task with a visual search task. The results showed that observers attended more to a stimulus they experienced as response-predictive in the preceding feature discrimination task. To examine to what extent selection history can be suppressed by goal-driven process, Kadel et al. (2017) tested different goal-driven-influenced modes of task preparations such as pretrial task cuing. As their results showed, attentional biases induced by selection history persisted despite task preparation. Their results show that even with these preparations, selection history still plays a noticeable role in biasing attention toward a formerly experienced target (see also Abbasi et al. 2022). Wolfe and Horowitz (2017) mentioned that not only the three aforementioned contributions but also the scene structure and the relative value of the targets and distractors must be considered in modern visual guidance theories. In Guided Search 6.0 (Wolfe, 2021)—the latest version of the Guided Search model on visual search and selective attention—these five factors are integrated in a spatial priority map to guide attention.

An integrated priority map was also proposed by Awh et al. as a theoretical framework for explaining how selection history and other factors of attention guidance interact (Awh et al., 2012; Theeuwes, 2019). Priority maps have been successfully employed by many authors (Fecteau & Munoz, 2006; Zelinsky & Bisley, 2015; Klink et al., 2014; Todd & Manaligod, 2017; Veale et al., 2017; Chelazzi et al., 2014) to explain the result of attentional priority and guidance in a visual scene. In a review, Klink et al. (2014) summarized how goal-driven and stimulus-driven maps in cortex combine with a value-based map in midbrain. This combination results in a priority map for the frontal eye fields. Zelinsky & Bisley (2015) speculated about the importance of a priority map in relationship with visual working memory and also with the motor system. They also highlighted this map as an appropriate construct for predicting behavior.

Stimulus-driven models of attention were developed early on Itti et al. (1998). These models tend to ignore the effects of selection history, task or training (Itti & Borji, 2015). Itti et al. (1998) implemented feature integration theory (three feature maps including color, intensity and orientation), the winner-take-all principle, inhibition of return and a normalization method to model visual attention in a stimulus-driven manner. This model (or its elaborated version; Itti & Koch 2000) was subsequently expanded (Ramirez-Moreno et al., 2013; Tanner & Itti, 2019). de Brecht & Saiki (2006) showed how Itti and Koch’s model (Itti & Koch, 2000) can be implemented by neural networks with biologically realistic dynamics based on data from electrophysiology experiments. This model was also expanded later by integrating motion saliency computation (Ramirez-Moreno et al., 2013). Itti’s stimulus-driven model was also combined in a goal-driven model (Tanner & Itti, 2019) to represent the effect of goal-relevant information on attention or eye movement. Veale et al. (2017) validated a neural implementation of Itti’s model. In another stimulus-driven model, Bruce & Tsotsos (2009)—using self-information maximization ($-\log (p(x))$), where *x* is a feature—proposed a computational model of saliency that is called “Attention based on Information Maximization (AIM),” because attention is attracted by surprising, i.e., potentially informative, regions of an image.

Most of the models reviewed so far were developed to explain data from highly controlled experiments with impoverished artificial stimuli. However, humans deploy their attention in uncontrolled natural settings replete with complex stimuli. Thanks to deep learning advances, there has been recent progress in deep visual saliency models that can process complex natural images (Borji, 2019). DeepGaze II is a saliency model that predicts where people look using features from a pre-trained convolutional neural network (VGG-19) and a few layers on top that are trained to read out saliency (Kümmerer et al., 2016). While these models have near-human performance compared to observers in front of a screen, they mostly explain saliency effects at their current state of development. It will be interesting to include other attentional guidance mechanisms in them, which go beyond the currently presented scene.

Itti and Borji reviewed more than 50 computational stimulus-driven models (Itti & Borji, 2015). Computational models that integrate goal-driven control (Navalpakkam & Itti, 2005; Hwang et al., 2009; Borji et al., 2014) are less well researched than saliency models, likely because they require information not available in the stimulus. Some models integrate stimulus-driven and goal-driven signals in attentional guidance (Kimura et al., 2008). Chikkerur et al. (2010) used a Bayesian framework to explain how a combination of stimulus-driven and goal-driven attentional guidance work together in cortex.

Previous studies showed that attention can be biased more toward a target feature which was selected in the last trial (Maljkovic & Nakayama, 1994; Kristjánsson & Campana, 2010; Theeuwes & van der Burg, 2011; Kadel et al., 2017). This effect, known as intertrial priming, is one of the lingering biases attributed to selection history (Theeuwes, 2018). Selection history has hardly been modeled despite being a well-known phenomenon. Tseng et al. (2014) implemented a Ratcliff-type diffusion model (Ratcliff, 1978) for a 2-forced-choice task and showed that intertrial priming can affect diffusion model parameters.

**Fig. 1:**
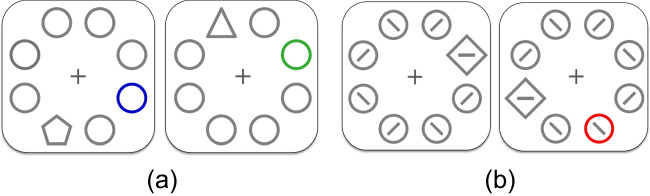
Experiment displays. Learning task (**a**): Participants in the color group had to respond to the color (green vs. blue) and participants in the shape group had to respond to the shape (pentagon vs. triangle). Search task (**b**): The orientation (horizontal vs. vertical) of the line embedded in the diamond had to be reported in distractor-absent (left) and distractor-present trial (right)

In this paper we introduce an algorithmic-level or “mechanistic” model (in the sense of Marr 1982) to show how stimulus-driven processes, goal-driven control and selection history compete to guide visual attention toward a specific target^1^. We operationalize selection history as the effect of training-phase learning on the test phase (see Feldmann-Wüstefeld et al. 2015; Kadel et al. 2017; Henare et al. 2020). The model comprises a priority map to integrate goal-driven, saliency-based and history-related biases in a winner-take-all manner. Stimulus-driven guidance, feature maps and saliency maps are made based on feature integration theory (Treisman & Gelade, 1980) and self-information maximization (Bruce & Tsotsos, 2009). Feature integration theory, developed by Treisman & Gelade (1980), posits that separable dimensions (such as shape and color) are processed separately before being integrated on-demand. Using this theory, the proposed model, codes the input into three types of features (color, shape and orientation) and computes a saliency map for each feature dimension using self-information maximization (Bruce & Tsotsos, 2009). Unlike saliency models which work on image pixels, we represent 1-out-of-K encoded maps, because we are primarily interested in the interaction between different attentional guidance mechanisms on a conceptual level.

The model also incorporates an intertrial effect which emphasizes the response-relevant feature dimensions (either color or shape in learning task and orientation in search task, see Fig. 4) of the last trial in the current one; Found & Müller (1996). Additionally, a history map contributes to the integrated priority map to reflect the effect of selection history and learning in the model. Finally, task-relevant information controls the map integration weights that generate predictions for responses and response times. These integration weights are our model for the goal-driven influences. We test this model on a behavioral database from an experiment by Feldmann-Wüstefeld et al. (2015). The model can predict the reaction time distribution parameters for each participant and also across the experimental groups. To find the best fitted distribution on reaction times, several probability density functions are compared minimizing negative log-likelihood and the best fitting one—an ex-Gaussian distribution (Matzke & Wagenmakers, 2009)—is used in the model.

The rest of the paper is organized as follows: we review the experiment and explain its details required for a full understanding of the model. We then compare models that differ in the information that enters into the integrated priority map and show that a model with selection history information—on feature level—performs best. We also show that the inclusion of intertrial effect variables leads to an increase of the (approximate) Bayesian model evidence. More information about reaction time distributions can be found in Appendices A and B. Besides, a general linear model is presented in Appendix C.

## Experimental Data

The data used in this study comes from the first experiment of Feldmann-Wüstefeld et al. (2015). They investigated the impact of associative learning on covert selective visual attention to examine whether selection history effects generalize from particular features (e.g., “blue” or “green”) to the entire color dimension. The experiment consisted of a “practice” and a “main” phase, in which two types of tasks (learning and search) were performed. A central fixation cross was presented on the screen, which was then surrounded by eight different elements on an imaginary circle (Fig. 1). 28 participants were divided randomly into two different groups, namely “color group” and “shape group.” They were first naive about their group membership, but had to learn it on a trial and error basis in the practice phase.

In the “practice phase,” participants had to learn that either color or shape was the response-relevant dimension in this learning task (see Fig. 1a). Members of the color group had to report the color of the color singleton (blue or green), whereas members of the shape group had to respond to the shape of the shape singleton (triangle or pentagon). They had to use their left hand to press one of two buttons that were placed on the left side of the response pad. Auditory feedback indicated whether they pressed the incorrect key.

In the “main phase” a second visual search task was added, and participants performed both tasks in random order. In the search task (Fig. 1b), all participants had to search for a shape target and report the orientation of a line presented inside the diamond-shaped target. In half of the trials, a response-irrelevant red circle was presented as distractor. Participants used their right hand to press one of two buttons on the right side of the pad to indicate the line orientation (horizontal versus vertical).

The results of this study showed that the history of selection acquired in the learning task affected the participants’ performance in the search task. Reaction time analysis showed that responses in distractor-present trials were slower than in distractor-absent trials, and the distractor cost was larger in color group participants than in participants in the shape group. Concurrently recorded EEG signals also suggested that participants in the color group deployed attention toward the red color distractor, this was not the case for participants in the shape group. Accordingly, the authors suggested that the participants’ history of either shape or color selection in the practice phase had resulted in a selection history bias. Feldmann-Wüstefeld et al. had done their study in 4 experiments to examine the influence of task switching. In the first and the second experiments, learning and search trials were intermixed. In experiment three, the tasks were presented block-wise and in the fourth one, the tasks were performed on separate days. The results of all experiments demonstrated the presence of selection history effect on attention deployment even when the tasks were done on different days (Feldmann-Wüstefeld et al., 2015). We decided to use the intermixed presentation trials to model the effect of repeating the response-relevant feature dimension from trial n-1 to trial n (intertrial effect) along with goal-driven, stimulus-driven and selection history influences on attentional control. We present a generalized linear model analysis of the effects of distractor presence and intertrial in Appendix C. This analysis confirms the presence of decisive evidence (in the sense of Kass & Raftery 1995) for both effects in the data.

We developed a model of this selection history bias in the current study based on the behavioral data from the main phase, which comprises a total of 28,672 trials across all participants. More details about the experiment can be found in Feldmann-Wüstefeld et al. (2015).

## The Algorithmic Model

**Fig. 2:**
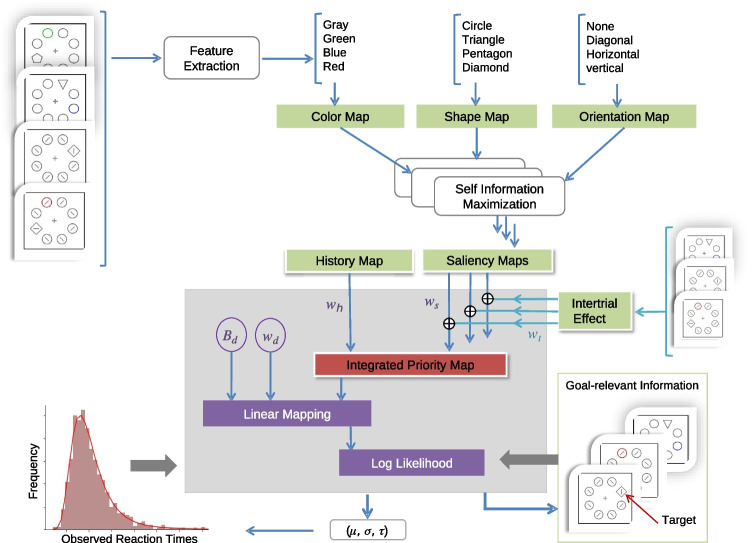
An overview of the algorithmic model. The blue arrows show the direction of data flow from visual input to response and gray arrows show the direction of feedback. $w_s, w_h, w_t$ and $w_d$ are model parameters that weigh how strongly different maps enter into the integrated priority map or the RT prediction. $w_s$ is saliency weight with three elements for color, shape and orientation. $w_t$ is intertrial effect weight and also has three elements for color, shape and orientation. $w_h$ is history map weight. $w_d$ is distribution parameters weight and has three elements for $\mu , \sigma$ and $\tau$. $B_d$ is distribution parameters’ bias containing $B_\mu , B_\sigma$ and $B_\tau$. The goal-relevant information (on the right side of the figure) helps the model to guide attention to the target location

We assembled an algorithmic-level model to explain how goal-driven and stimulus-driven influences competitively interact with visual selection history to guide attention toward a specific stimulus. Inspired by the integrated priority map in Awh et al. (2012), we used a “history map” reflecting the influence of selection history on current attention deployment, see Fig. 2. Additionally, there is an overall saliency map for stimulus-driven influences. How these maps combine into an integrated priority map is determined by task-dependent weights. Figure 2 also shows how the output of the integrated priority map is used to predict ex-Gaussian distribution (Luce, 1986) parameters of reaction times (left exit path in the figure) and response likelihoods (the right exit path). Evaluating these response likelihoods and reaction times against participants’ reaction times allows us to fit the model to the experimental data, see Eq. 5 below. Since the model employs the ex-Gaussian as an RT distribution, we detail in Appendix A how this distribution was chosen based on a model comparison.

**Fig. 3:**
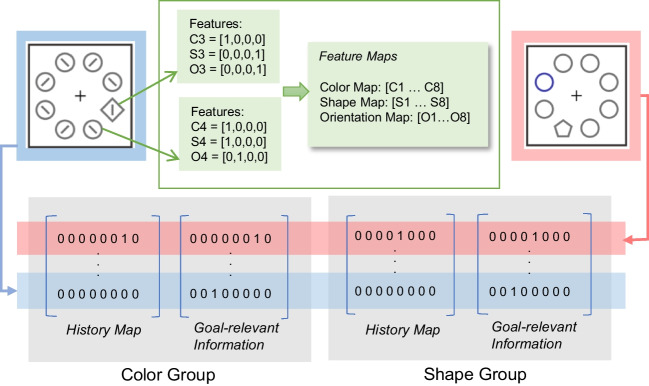
Feature maps, history map and goal-relevant information for two random trials. We use 1-out-of-K encoding for the feature vectors, i.e., all components but one are zero. The nonzero component indicates the feature value (see the green box). In each row of history map the location of learned feature is marked. In goal-relevant information the location of the response-relevant feature is marked

The input stage of the model is based on assumptions made by visual search theories such as feature integration (Treisman & Gelade, 1980) and guided search (Wolfe, 2021). The model extracts three types of features (color, shape and orientation) and feature maps—as shown in Fig. 2—are computed. In the next processing step, saliency maps that model the effect of stimulus-driven control on visual attention (Koch & Ullman, 1985) are derived from the feature maps by computing Shannon’s Self-Information on the feature statistics. Our approach is therefore related to Attention based on Information Maximization (AIM) (Bruce & Tsotsos, 2009). However, in AIM self-information is computed from features extracted from the image pixels, rather than our predefined features. We chose predefined features due to the stereotypical nature of our stimulus images. Equations 1 and 2 show the actual calculations behind map computation. Feature maps are $M\times N\times K$ vectors where *M* is the number of trials, *N* is the number of objects in each trial and *K* is the number of distinct values that each feature can take on, i.e., we are using 1-out-of-K encoding for the features, with the value 1 indicating which feature value is present. In the current experiment $M=1024$ (for each participant), $N=8$ and $K=4$. Figure 3 illustrates the method of building feature maps for some example trials. For all trials, we take the feature maps $f_i$ for $i \in \{color, shape, orientation\}$ and compute the self-information $X_i$:

1 $$\begin{aligned} \forall k: X_{i}[k] = -\log \bigg (\sum _{n=1}^{N} f_{i}[n][k]/N \bigg ) \end{aligned}$$

which yields the saliency of all trials $s_i[n]$:

2 $$\begin{aligned} \forall n: s_{i}[n] = X_{i}\left[ \underset{k}{\text {arg}\,\text {max}}( f_{i}[n][k] ) \right] \end{aligned}$$

where, due to the 1-of-K feature encoding, we can use *argmax* to pick the self-information corresponding to the current feature value.

Saliency maps $s_i$ are fed into the integrated priority map along with history information (*h*) to compete in a softmax model for the predicted response target. Selection history, the third category of attentional guidance (Awh et al., 2012), carries the effect of learning (participants learned about color or shape in our experiment) into the priority map (*p*).

To model the intertrial effect on participant’s RT, we added another parameter ($w_t$) to the model. The parameter $w_t$ includes a weight for each feature dimension (correspond to Dimension Weighting Account; Liesefeld et al. 2019) and it modulates the saliency weights when the maps are combined into the priority map:

3 $$\begin{aligned} \forall m,n: p[m][n] = \underset{n}{\text {sof}\,\text {tmax}} \bigg ( \sum _{i}\big ( (w_{s_i} + w_{t_i} * t_i[m]) \\ * s_{i}[m][n] \big ) + w_h*h[m][n] \bigg ) \nonumber \end{aligned}$$

**Fig. 4:**
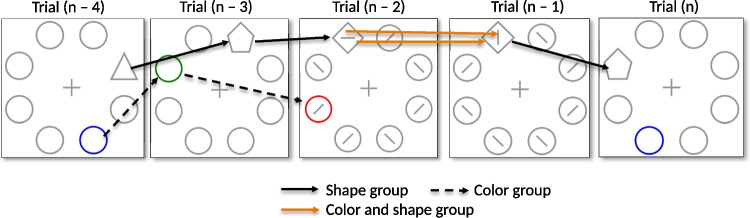
Intertrial effect. This effect in our model is depicted by solid lines for shape, and dashed lines for color. Attending a particular feature (e.g., triangle shape in trial n-4) primes shape dimension attention in the next trial, here: pentagon in trial n-3. Likewise for color dimension. Line orientation priming is possible too, shown for trial n-2 $\rightarrow$ trial n-1 (upper orange arrow). Note that shape priming is possible in the color group too if two search trials follow each other (lower orange arrow)

The weights ($w_h$ for history and $w_{s_i}$ for $i \in$ {*color, shape, orientation*}) are used to combine the history map and the saliency maps computed from color, shape and orientation. These weights reflect the influence of the content of the respective map on the integrated priority map for the tasks that the model will be optimized for. In Eq. 3*t* is a $M \times i$ matrix which carries information from the last trial: in each row of *t*, a “1” indicates the feature dimension which had to be selected by the participants in the last trial (see Fig. 4). The softmax function is used to ensure that the winning location receives the most attention while keeping the map interpretable as a probability distribution.

In our model, Eq. 3 can be interpreted as the first layer of a (two-layer) neural network. The second layer is a (linear) mapping from the integrated priority map to reaction time distribution parameters:

4 $$\begin{aligned} \forall m: d[m] = \sum _{n=1}^{N}(p[m][n]*w_d)+B_d \end{aligned}$$

where *w* and *B* are weights and biases of ex-Gaussian distribution parameters’ $d[m] \in (\mu [m], \sigma [m], \tau [m])$ for each trial *m*.

We also compute a 1-out-of-K representation of the goal-relevant information (*g* in Eq. 5) (see Fig. 3) which is used for machine-learning the weights with which the history map and the saliency maps are combined in the priority map. Psychologically, we can interpret the role of this as combined guidance of stimulus-driven and history toward the location of the target.

The weights and biases, which comprise the model parameters $\theta =(w_h, w_{s}, w_{t}, w_d, B_\mu , B_{\sigma }, B_\tau )$ for both tasks are determined by minimizing the loss function. This function comprises the negative logs of the joint RT distributions, the goal-relevant information (*g*) under the distribution predicted by the integrated priority map (*p*) and eventually the prior distributions over the model parameters $p(\theta )$:

5 $$\begin{aligned} Loss= & {} - \sum _{m=1}^M \log \big (ExG \big ( RT[m]\,|\,\mu [m], \sigma [m], \tau [m] \big ) \big )\\- & {} \sum _{m=1}^{M} \sum _{n=1}^{N} \big ( \log p[m][n] * g[m][n] \big ) - \log p(\theta ) \nonumber \end{aligned}$$

where ExG is the ex-Gaussian density function. The prior distributions are:

6 $$\begin{aligned}&w_{h|s|t|d}\sim \mathcal {N}(0.0,1.0) \nonumber \\&B_\mu \sim \mathcal {N}(600.0,100.0) \nonumber \\&B_{\sigma } \sim \mathcal {N}(75.0,4.0) \\&B_\tau \sim \mathcal {N}(200.0,20.0) \nonumber \end{aligned}$$

when the mean and the standard deviation of the last three distributions are selected in a way that matches results from similar experiments (Feldmann-Wüstefeld et al., 2015; Kadel et al., 2017).

**Fig. 5:**
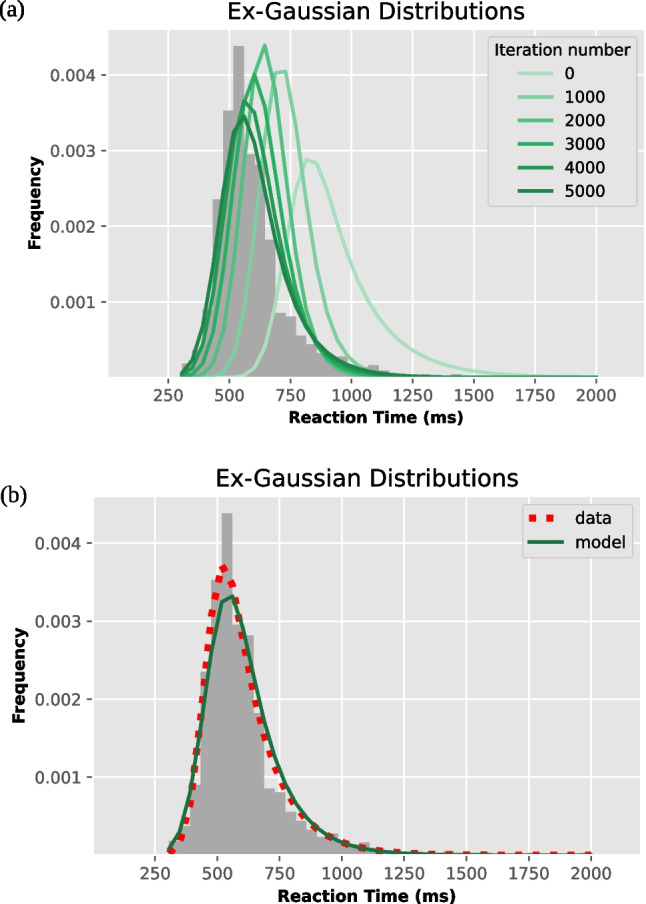
Ex-Gaussian distributions of a participant’s reaction times. (**a**) The model-predicted distributions are plotted every 1000 iterations of the model optimization. At iteration 0, the optimization begins with parameters randomly drawn from the priors. The darkest plot (after 5000 iterations) shows the best fit. (**b**) Best model-free fit to the data (red) and model-predicted distribution (green). For more examples please refer to Appendix B

To find the weights and biases that minimize the loss (Eq. (5)), we draw random initial values from these distributions (Eq. (6)) and then optimized using Python 3.8.8, PyTorch 1.8.1 and Adam optimizer with learning rate 0.1. In each optimization step, maps’ weights (e.g., $w_h, w_s$) and the reaction time distribution parameters ($\mu , \sigma , \tau$) are updated to reach to the best possible distribution fit on the data (see Fig. 5a). The model approximates the reaction time distribution parameters very well (as can be seen in Fig. 5b). To quantify how close the model-predicted distributions are to the best fit to the data, we evaluate an approximation to the Kullback-Leibler (KL) divergence (Bishop, 2006):

7 $$\begin{aligned} KL(p||q)= \int p(RT)\log \big ( \frac{p(RT)}{q(RT)} \big ) ~dRT\\ \approx \frac{1}{M} \sum _{m=1}^{M} \log p(RT_m) - \frac{1}{M} \sum _{m=1}^{M} \log q(RT_m) \nonumber \end{aligned}$$

where $RT_m$ is the reaction time in trial m, p(RT) and q(RT) are model-predicted and best-fit distributions respectively. For both color and shape group RTs, we find $KL(p||q) \le 10^{-4}$ which is very close to the minimal possible value of zero.

## Results and Discussion

To investigate how selection history and saliency maps quantitatively predict attentional guidance, we tested seven versions of the model. Table 1 summarizes these versions. In the first model (M1), the history map contains the response-relevant features of the learning phase (blue and green for the color group, triangle and pentagon for the shape group). This model is used as the basis for models M2 to M7, which are altered versions thereof. In M2 the history map includes all color singletons (for participants in the color group) and all shape singletons (for participants in the shape group). Here, the assumption is that the participants have learned response predictiveness on the dimensional level (color or shape), not on the level of single features (such as green or blue). So not only blue, green, triangle and pentagon but also red and diamond are included. In M3, priming information from previous trials is removed from the model. In M4, we exclude the history map from the model testing the assumption that only goal-driven and stimulus-driven guidance direct attention. In M5, M6 and M7, shape, color and orientation maps are removed to see if all feature maps are needed to guide attention in this experimental paradigm.

To compare the versions of the model, we use a Laplace approximation to the Bayesian model evidence (Bishop, 2006; Barber, 2011; Endres et al., 2013):

8 $$\begin{aligned} LAP= & {} \underbrace{\log ( p (D|\hat{\theta },Q) )}_\text {log-likelihood} + \underbrace{\log (p(\hat{\theta }|Q))}_\text {log-prior}\nonumber \\{} & {} - \underbrace{\frac{1}{2} \log (|H|) + \frac{L}{2} \log (2\pi )}_\text {log-posterior-volume} \end{aligned}$$

where $\theta$ is the vector of parameters for each model (*Q*), $L=\text{ dim }(\theta )$ is the number of parameters and $\hat{\theta }$ is the value of $\theta$ at the mode of the posterior distribution. *H* is a Hessian matrix: a $L \times L$ matrix of the second-order derivatives (of the negative log-posterior) evaluated at $\hat{\theta }$, and |*H*| denotes the determinant of *H*.

As shown in Eq. 8, the Laplace approximation (LAP) consists of three components. The first component (log-likelihood) measures the goodness of fit and is defined as:

9 $$\begin{aligned} \log ( p (D|\hat{\theta },M) ) = \underbrace{\sum _{m=1}^M \log \big (ExG \big ( RT[m]\,|\,\mu [m], \sigma [m], \tau [m] \big ) \big )}_\text {reaction time log-likelihood}\\ + \underbrace{\sum _{m=1}^{M} \sum _{n=1}^{N} \big ( \log p[m][n] * g[m][n] \big )}_\text {attention log-likelihood} \nonumber \end{aligned}$$

which is also part of our loss function (see Eq. 5 and the text below that for more details). The third component (log-posterior-volume) of Eq. 8, measures how well the data constrain the parameters. The sum of this component and log-prior, which is known as “Occam’s razor” (Bishop, 2006), penalizes the complexity of the model and guarantees that models with more parameters do not score better by simply overfitting the data.

**Table 1:** A summary of the model versions

Models	Shape map	Color map	Orientation map	History map	Intertrial effect
M1	$\surd$	$\surd$	$\surd$	Featural	$\surd$
M2	$\surd$	$\surd$	$\surd$	Dimensional	$\surd$
M3	$\surd$	$\surd$	$\surd$	Featural	–
M4	$\surd$	$\surd$	$\surd$	–	$\surd$
M5	–	$\surd$	$\surd$	Featural	$\surd$
M6	$\surd$	–	$\surd$	Featural	$\surd$
M7	$\surd$	$\surd$	–	Featural	$\surd$

A featural history map contains all response-relevant features in the practice phase

A dimensional history map contains all the features related to the learned dimension

**Fig. 6:**
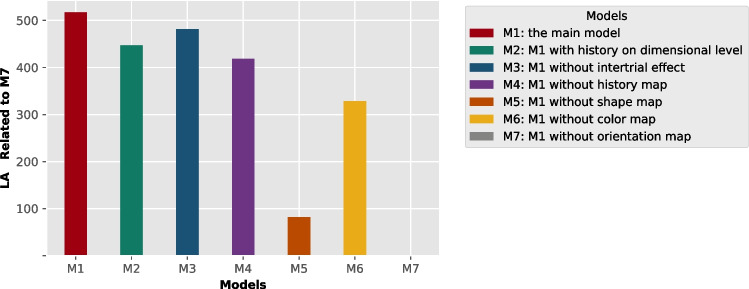
Model comparison. We computed a Laplace approximation to the Bayesian model evidence across all participants (see LAP, Eq. (8)). The LAPs are plotted relative to M7 (the least probable model). Bigger LAP is better. M1, which is called the main model, contains the saliency maps, intertrial effect and the history map on response-relevant features. This model scores best. For models descriptions, see the text

**Fig. 7:**
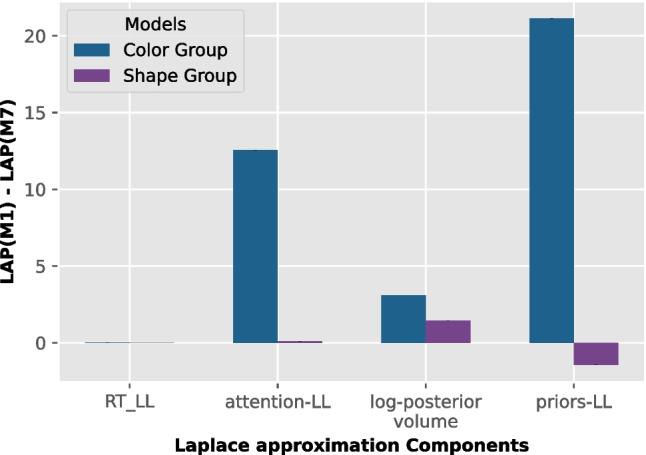
Model comparison based on Laplace approximation (LAP) components. The components are shown on x-axis: RT log-likelihood (LL), attention log-likelihood, log-posterior-volume and priors log-likelihood. The details about the components can be found in Eqs. 8 and 9. The difference between the most and the least probable models (M1 and M7, relatively) are shown on the y-axis. For each model we calculated the mean of the model evidences (LAP values) for all participants in a specific group (either color or shape group). Error bars are very small and cannot be seen

In our model comparisons, we first use LAP to find the best version of the model. Then we look at the LAP components to see what exactly causes a model to score better than the others. The model evidences (LAP values) for seven versions of the model are shown in Fig. 6. These models are described at the beginning of this section and are also summarized in Table 1. Each model version is fit individually to each participant; then, LAP scores are added per model version. The results show M1 being the most probable model. Please note that the values on Fig. 6 are $\log _e$ probabilities. So for instance since the difference between M1 and M3 (the second best model) is about 20, M1 is approximately $10^{8}$ times more probable than M3. The M1 model includes saliency maps for color, shape and orientation, a history map—with the features that were predictive during learning—and also the effect of the last trial on the current one (i.e., an intertrial effect). By excluding the orientation map from M1 we get the least probable model (see M7 on Fig. 6). To find out what causes this big reduction in model evidence, we look at the LAP components: reaction time log-likelihood, attention log-likelihood, log-prior and log-posterior-volume. The bar charts in Fig. 7 indicate the difference between M1 and M7 regarding LAP components, separately for color and shape group participants. The first component (reaction time log-likelihood) indicates which model predicts RTs and their distribution parameters better. The results show all models are equally accurate in predicting these parameters. On the other hand, the second component (attention log-likelihood) indicates how well the model predicts target locations. If we compare M1 and M7 regarding this component, M1 shows a much better performance in color group participants.

**Fig. 8:**
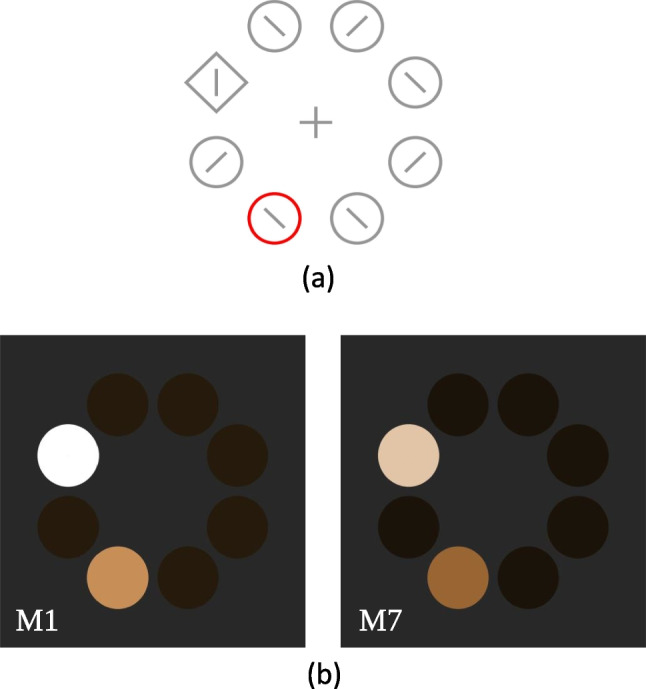
Attending location, predicted by the models M1 and M7 trained on a color group participant. (**a**) Stimulus of a distractor-present trial. (**b**) Integrated priority map for both models for a color group participant. The brighter the colors are, the more likely it is that attention is deployed to a location. M1 makes a better prediction of attention being correctly deployed to the target location

The log-posterior-volume terms are comparable in both groups, indicating that the parameters of all models in all groups are similarly well constrained. The log-prior terms differ substantially between M1 and M7 in the color group only. This is due to M7 having to put a large weight on the featural selection history map to guide attention away from the color distractor. Such a large weight is improbably under the prior (see Eq. 6), making M7 less likely a posteriori. Yet, even with this large weight, M7 is a worse explanation of participants’ behavior, as can be seen by the difference in the attention-LL terms. This is illustrated in Fig. 8: M1 is a better predictor of a color group participant’s attention guidance to the correct target location. We interpret this result as evidence for the hypothesis that orientation can be used to deploy attention, too.

In addition, we would like to point out that the potential effect of orientation on attentional deployment arises from the way in which our models integrate individual (saliency/history) maps into an overall priority map. Since in our experimental data the orientation is unique (either horizontal or vertical) and at the same location with diamond-shaped target, we have at this point no independent causal evidence for or against this model-based hypothesis, as pointed out by one of our reviewers.

**Fig. 9:**
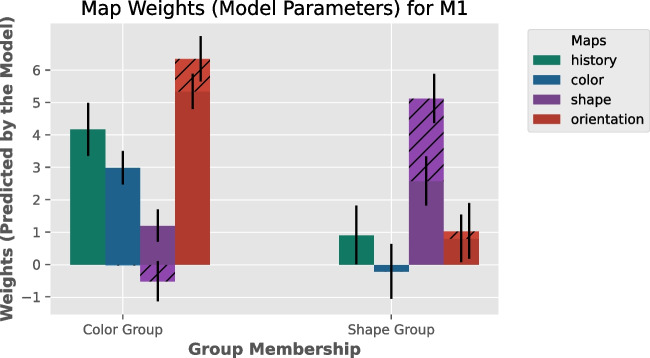
Map weights. Optimal map weights for the first model are shown for the color and the shape group. A higher weight means a stronger influence of the corresponding map onto the response and reaction time. The hatched parts are the weights modulations by the intertrial effect. Note that the final weight of each saliency map is a sum over the map weight and the priming weight, see Eq. 3 and also Fig. 2. Priming modulations on color maps are very small (close to zero) and can be hardly seen. Priming modulation on shape map in color group is negative. The error bars represent the standard deviations of the posterior, i.e., standard errors

**Fig. 10:**
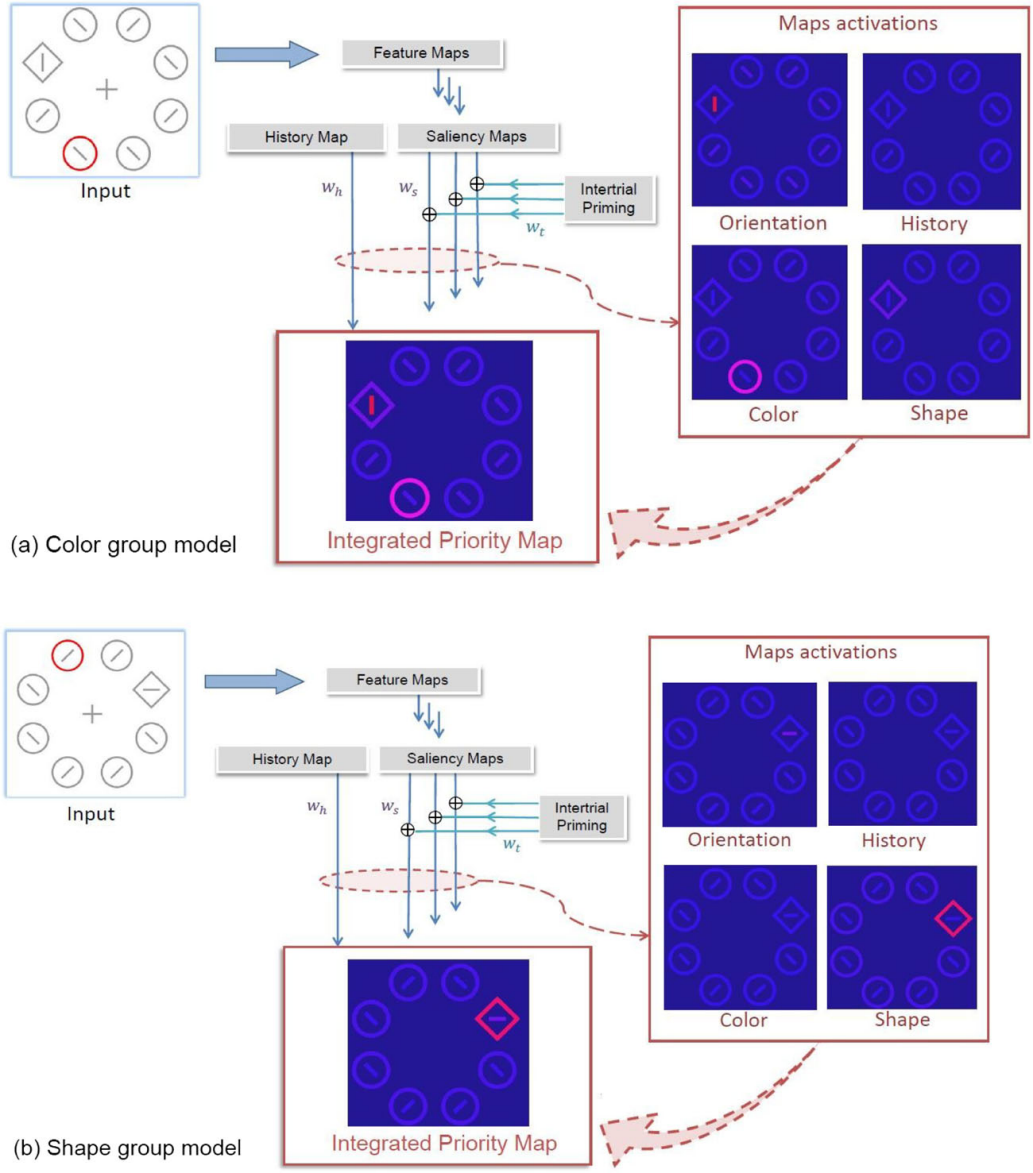
Maps activation in distractor-present search task trials for a color group (a) and a shape group (b) participant model. To visualize this activation the weighted value of each map is used as a color code: ($(w_{s_i} + w_{t_i}) * s_i$) for each saliency map (*i*) and ($w_h * h$) for history map. See Fig. 2 for variable names. Warmer colors indicate higher activation. Individual weighted maps activation is integrated in the final priority map and attention is guided to the location with the highest feature activity. See Eq. 3 for a computational description

Under the assumption that there is at least an approximately linear mapping from the priority map to the reaction time distribution parameters, the model machine-learns to predict the history map weight ($w_h$), saliency map weights ($w_s$), intertrial effect weights ($w_t$) and also the distribution parameters weights and biases ($w_d$, $B_d$) (see Fig. 2). A comparison of the learned weights and their differences between the color group and the shape group is shown in Fig. 9. As one might expect, the color weight is higher in the color group, whereas the shape weight dominates in the shape group. This leads to a stronger influence of the respective saliency map on the contents of the integrated priority map, which is shown in Fig. 10 for a distractor-present trial. In other words, while we assume that saliency is a property of the physical stimulus statistics, the weight with which saliency enters into the integrated priority map can be varied by (learnable) task demands. In Fig. 10 the individual map activations and their weighted combinations are shown in color coding. The color group model’s attention is strongly drawn toward the (red) color distractor. In contrast, the shape group model prioritizes the correct target location.

As Fig. 9 shows, the “history map” has a higher weight ($w_h$) in the color group than in the shape group: to solve the learning task, the color group model has to rely on the colors (blue and green) encountered during the practice phase which is reflected in the large weight of the history map. Although these colors could be found in the “color map” as well, there is another color (red) in this map which is task-irrelevant and has to be suppressed, hence the smaller weight of the color map. This is the reason for the increased attention capture by the red distractor in color group members which is reported in Feldmann-Wüstefeld et al. (2015). In other words, the presence of a color distractor leads to a down-weighting of dimensional color saliency in favor of a feature-level color representation. For the search task, a high orientation weight is employed by the color group model, since orientation can potentially influence attention deployment. Please see the discussion below in this section.

In contrast, the shape group model can afford to rely mostly on the “shape map,” because the items in its history (triangle and pentagon) exist in the “shape map” too (triangle, pentagon and diamond), and there is no shape distractor. Therefore, by using a high shape map weight, both the learning task can be solved and attention can be guided to the shape singleton containing the target in the search task (diamond).

To summarize, the weight of the “orientation map” is larger in the color group than in the shape group, indicating that the color group model relies on orientation saliency in the search task. However, the shape group model focuses on the “shape map” which is response-relevant in both tasks. Also, the weight of the “color map” was higher in the color group than in the shape group model, since the latter group can ignore color altogether.

As it is highlighted earlier (see Fig. 6), the model evidences fall down when any of the feature maps are excluded from the model. By that we claim all feature maps are needed to have a better prediction on the locations of the targets. This observation is more significant when the orientation map is excluded (M7 in Fig. 6). A closer look at the model comparison method (see Fig. 7) confirms this finding—specifically for the color group participants. The model also predicted a higher weight for the orientation map than the other maps in the color group (see Fig. 9). One might claim that what we have reported here regarding the role of the orientation in guiding attention contradicts with the design and the main assumption of the additional singleton paradigms.

Additional singleton paradigms (Theeuwes, 1991) have often been used to investigate how selection history effects—such as reward (Anderson et al., 2011), learning (Feldmann-Wüstefeld et al., 2015) or predictability of distractor location (Wang & Theeuwes, 2018)—alter visual attention. In the additional singleton paradigm, participants are confronted with search displays where the target is defined in a particular dimension (e.g., shape) and an additional distractor, salient in a different dimension (e.g., in color), is presented in some of the trials. Crucially, the search-relevant and the response-relevant features differ; the response-relevant is neither defined in shape nor in color, but it is a line orientation embedded inside a display item that indicates which response key has to be pressed. The idea behind using such a compound task was to keep the response-related, post-selection processing parts separate from those processes that relate to attentional selection processes. Having that said, more recent literature has proven the difficulty of separating pre- from post-selective processes. When considering the target template that an observer needs to maintain as a representation of the task-relevant features in working memory (e.g., Geng & Witkowski 2019; Tünnermann et al. 2021), orientation seems a likely candidate to be part of this representation as well. This does not imply that orientation is a feature of similar importance as shape to guide attention to the target item, but orientation is definitively needed to accomplish the task.

There has been considerable debate regarding whether participants can successfully perform the task by focusing solely on orientation while attempting to disregard color and shape information. Theeuwes argued that this might not be feasible, contending that participants cannot pre-attentively identify orientations (Theeuwes, 2010, 1991). However, the results of another study suggest that participants might indeed be capable of more swiftly identifying the response-relevant orientation by ignoring colors or shapes (Wu et al., 2019). This finding contradicts the conclusions drawn by Theeuwes (1991), underscoring the need for further research to gain a deeper understanding of how attention operates within the additional singleton paradigms.

Based on the model results, the response-relevant feature (the line embedded in the diamond-shape target) could potentially influence attention deployment if it partook in saliency processing and entered into the priority map. This assumption can be examined from (at least) two different perspectives:

On one hand, the line embedded in the target was of unique orientation in the display. Consequently, it might have contributed to selection via saliency-driven processing, at least to some extent. This is what we concluded from our modeling results. On the other hand, the model primarily emphasizes guiding attention to the target’s location, and the decision-making stage is not explicitly modeled. Therefore, the effect that we observe and interpret as the role of orientation in guiding attention could potentially be part of a response preparation process that initiates before attentional selection was completed. Given our reliance on behavioral data, it is important to consider that, in addition to attentional selection, other processes such as decision-making might have contributed. Total reaction time represents an accumulation of various components, and these components may overlap at certain points.

Another crucial aspect to highlight is the role of intertrial effect. To see how intertrial effect is defined in our model, please refer to Fig. 4. Our assumption is that intertrial effect is dimension-specific rather than feature-specific. This is also claimed by Liesefeld et al. (2019). In our model, intertrial effect has three weights ($w_t$) for color, shape and orientation. See also Eq. 3 and the hatched parts in Fig. 9. In the color group the modulatory intertrial effect causes an increased orientation map weight and also a reduction of the shape map weight. The former indicates that the generally high reliance of a color group model on orientation during a search task is amplified during repetition of search task trials. The latter might represent task switching: switching from the search task (reporting orientation embedded in a shape singleton target) to the learning task (reporting colors and not shape singletons) is best accomplished by down-weighting shape features temporarily. Interestingly, for color group participants, there is no intertrial-driven weight modulation of color map. Irrespective of the previous trial’s type, a color group model relies more on the history map than on the color map to ignore the red distractor. In our opinion, this rules out the alternative hypothesis that longer response times in the color group are induced by task switching efforts only, and not by selection history and the need to suppress the red distractor. This is in agreement with the results of experiments 3 and 4 reported in Feldmann-Wüstefeld et al. (2015). In both of these task variants, learning and search tasks were separated, either block-wise, or by asking participants to perform the tasks on separate days. Search performance of the color group, however, was still affected by their prior selections in the learning task, even though participants now performed only search tasks trials, and task switching no longer occurred.

## Conclusion

The presented model shows how saliency, selection history and goal-driven demands collaborate in guiding visual attention. The model implements the idea that selection history plays an important role in attention guidance as claimed in Feldmann-Wüstefeld et al. (2015). We compared different versions of the model and the results show that the one which includes selection history (long-learned selection preferences and also intertrial effect), besides stimulus-driven and goal-driven control, is best suited for a quantitative description of the behavioral (RT) results.

Visual attention modeling may have various purposes, such as image classification (Mnih et al., 2014), computer vision and robotics (Hiruma et al., 2022) or studying a specific experimental observation (which was also the goal of this paper). Consequently, as Tsotsos & Rothenstein (2011) mentioned, comparing models does not seem straightforward, fair, or useful so it might be better to compare some parts of the models which have relevant functionalities. The presented model in this paper, is similar to GS 2.0 in the way that it describes attention as a result of bottom-up and top-down activation in the priority map. Unlike GS which is a descriptive-level model, the presented model is on algorithmic level. An algorithmic-level model is a combination of descriptive models, mathematics and data fitting (Tsotsos & Rothenstein, 2011). In GS 6.0 (Wolfe, 2021), more attentional factors (reward, scene information and prior history) are taken into consideration. Clearly, a model with more mechanisms will give a better chance to move toward future naturalistic models since attention in the real world is influenced by many factors and not just saliency. We have already built the model composing three factors. Including more mechanisms in this model requires future experimental and modeling plans.

This paper is our first effort to model selection history as an attentional mechanism. To make the model more comprehensive, we plan the following future steps: since previous experiments on selection history effects were done with impoverished stimuli and simple participant responses, we planned to run an experiment in natural or semi-natural (virtual reality) environments. The data obtained in richer environments will likely require an extension of the model, in particular with respect to stimulus representation and response capability.

To construct a comprehensive computational model of visual attention, several interconnected modules must be developed. At a minimum, we require the implementation of early vision, scene understanding, priority mapping, and decision-making components (Wolfe, 2021). While we acknowledge the significance of modeling the decision-making component, the focus of this study was on modeling the priority map. The priority map furnishes essential information for the decision-making process. The decision-making process has been frequently modeled using a diffusion process over the past several decades (Wolfe, 2007; Moran et al., 2013). In future research, our objective is to integrate these components to create a more comprehensive model that can guide attention in natural(istic) settings, drawing on previous proposals (Wolfe, 2007; Moran et al., 2013; Schwarz & Miller, 2016; Allenmark et al., 2018). Furthermore, we intend to develop a dynamical version of the priority map that supports decision-making in changing environments, rather than responding to static stimuli. Importantly, our model does not yet include an explicit reinforcement learning component. Participants did learn the tasks from negative reinforcement only. Our model captures participants’ behavior after this reinforcement learning phase is completed, which was determined by a high enough performance level (see Feldmann-Wüstefeld et al. 2015). It would be interesting to model this first phase in future work, too. Another interesting avenue of investigation, which would help in constraining the model, would be the addition of physiological variables. For example, adding EEG signals to disentangle processes of target selection and distractor suppression would shed further light on attentional guidance processes.

## Figures and Tables

**Fig. 11:**
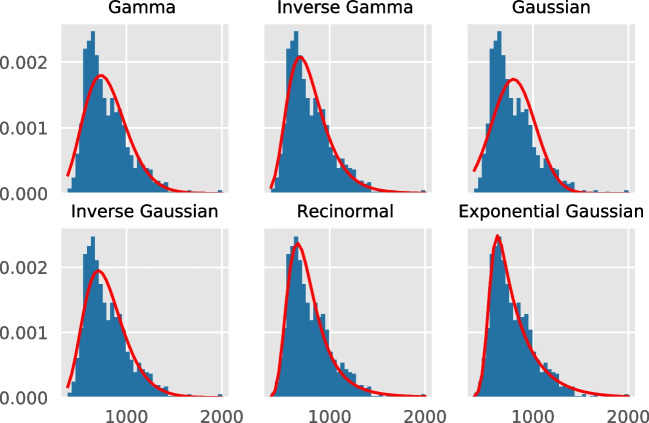
Distribution functions fitted on RT datasets for a random participant. Ex-Gaussian fits best

**Fig. 12:**
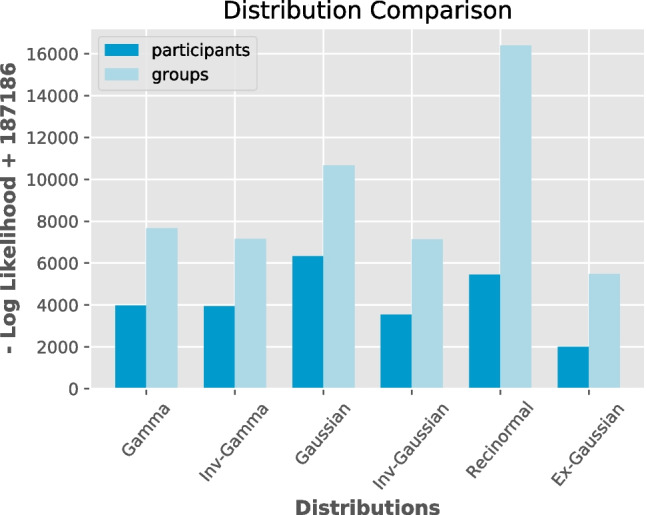
Distribution comparison using −log-likelihood score, lower is better. Distributions are fitted on individual participant’s data and on group-based data (all color group participants and all shape group participants). The best distribution is Ex-Gaussian, followed by inverse Gaussian

**Fig. 13:**
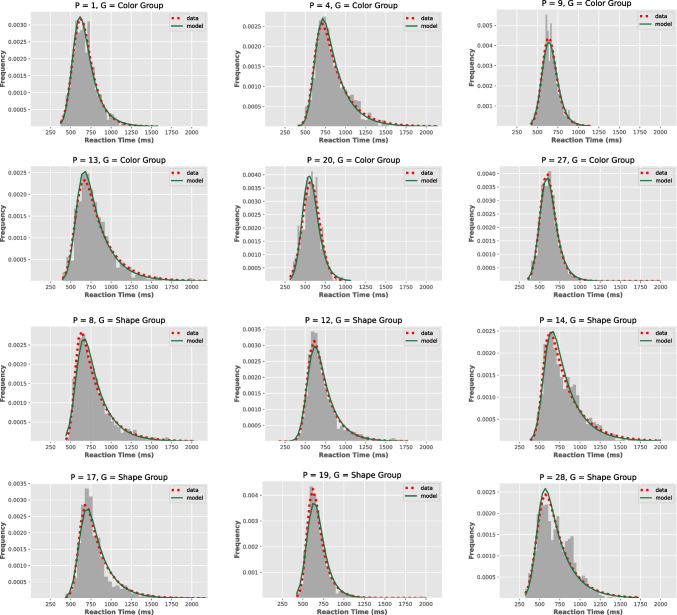
Ex-Gaussian distributions of 12 participants’ reaction times. Best model-free fits to the data are shown in red and model-predicted distributions are shown in green. This figure is an expansion of Fig. 5. “P” is participant number and “G” is group membership which is be either “color” or “shape” based on the participant’s learning experience. Note that all versions of the model are equally good in predicting the distributions

**Fig. 14:**
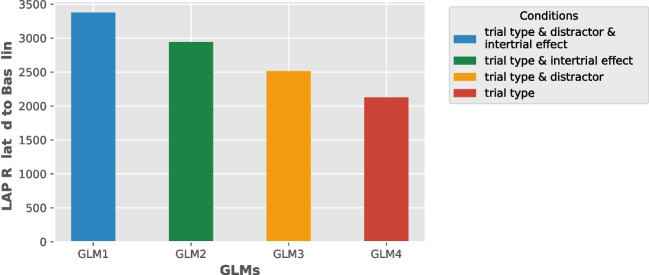
GLM comparison by Laplace approximation (LAP) to the logarithm of the model evidence, for different condition indicator combinations. LAP is relative to baseline model without any condition indicators. The best GLM accounts for trial type, distractor presence and intertrial effect

**Fig. 15:**
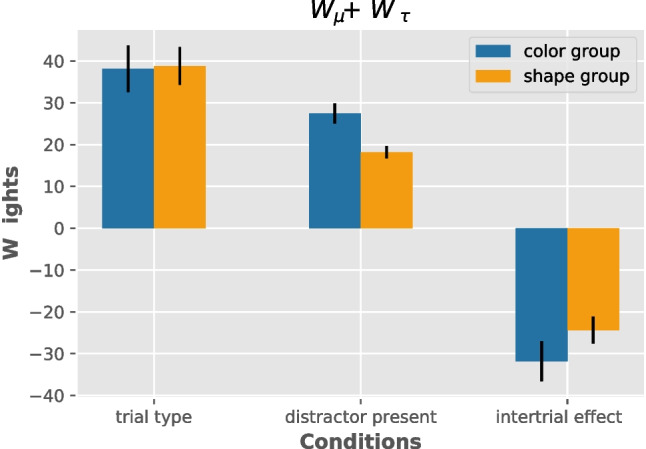
The influence of trial type, distractor presence, and intertrial effect on the mean of the RT distributions in GLM1. We fitted one model per participant, computed the weights and reported the mean of the weights per group. The error bars represent the standard deviation across participants within a group

## Data Availability

A preliminary version of codes and data are published here: http://dx.doi.org/10.17192/fdr/64.2. This repository will be updated if the paper is accepted.

## Appendix A: Reaction Time Distribution

Reaction time measurements have been widely employed in psychological experiments to analyze behavioral responses to well-defined tasks. Psychologists agree that there are three main types of reaction times: simple reaction times, recognition reaction times, choice reaction times and also some more forms such as discrimination reaction times and decision reaction times that come from combining varieties of experimental tasks (Harald Baayen & Milin, 2017).

Many distributions have been used to describe RT in neurocognitive and psychological research. In Santhanagopalan et al. (2018), the Gamma distribution is used to model PEBL (Psychology Experiment Building Language) Go/No-Go tests, with the primary motivation that RTs can be modeled better with a right-skewed distribution. In another study, inverse Gaussian (Wald) is used in a theoretical analysis of psychophysical parameters in a 2AFC design (Stone, 2014) with the assumption that if RT is the time needed for an evidence accumulation to reach a fixed boundary—similar to Brownian diffusion process—it is distributed as an inverse Gaussian. Another popular distribution is the Recinormal (Martin & Fermin, 2008) which is introduced in LATER model to describe psychological decision-making processes (Carpenter, 1981; Noorani & Carpenter, 2016).

We tried to find the best fitting distribution model for the reaction times in our data by testing the following distribution types against each other by approximate Bayesian model comparison: Gaussian, Gamma, inverse Gamma, inverse Gaussian, Recinormal and exponential Gaussian (ex-Gaussian). We fitted the distributions on each participant’s RT and also on aggregates defined by group memberships and trial types. We then compared the negative log-likelihoods, which showed that the best fit is achieved with an ex-Gaussian. For illustration, Fig. 11 shows the different densities (red lines) fitted to one of the participant’s RT data (histograms). Visual inspection indicates that the ex-Gaussian provides the best fit. For a quantitative comparison we computed each distribution’s parameters that minimized the sum of negative log-likelihood scores (participants-based and also group-based). These scores are depicted in Fig. 12, lower is better. The best fits are ex-Gaussian and inverse Gaussian models respectively. To fit the distributions on data we used Python 3.8.8 and PyTorch 1.8.1.

The ex-Gaussian distribution is a convolution of Gaussian and exponential distributions, see, e.g., Luce (1986). It has three parameters: $\mu$, $\sigma$ and $\tau$ that are the mean and standard deviation of the Gaussian component and the mean of the exponential component, respectively. The mean and the variance of this distribution are $\mu$+$\tau$ and $\sigma ^{2}+\tau ^{2}$. Equation 10 shows the probability density function (Moret-Tatay et al., 2018) where erfc is the complementary error function:

10 $$\begin{aligned} f(x)=\dfrac{1}{2\tau }e^{\frac{1}{2\tau }(2\mu +\frac{\sigma ^{2}}{\tau }-2x)}\text{ erfc }(\dfrac{\mu +\frac{\sigma ^{2}}{\tau }-x}{\sqrt{2}\sigma }) \end{aligned}$$

Response times are not distributed normally (Whelan, 2008). Because of their long tail on the right, RT distributions might have an exponential component. Christie & Luce (1956) and also McGill (1963) therefore proposed that RT distributions are a convolution of two components. For an ex-Gaussian distribution to arise, one of them has to have an exponential distribution. The above-mentioned authors had opposite beliefs about the source of this exponential component: Christie and Luce mentioned that decision time is exponentially distributed but McGill related that to movement response. Hohle (1965) also tried to show that the RT distribution is a convolution of normal and exponential components by auditory RT experiments.

The ex-Gaussian has been popular recently in psychological research. In Palmer et al. (2011) several distributions are fitted on behavioral data of three visual search tasks. The best fits all have an exponential component and the ex-Gaussian is one of those. Ex-Gaussian parameters can even be useful in evaluating attention disorders (Hwang-Gu et al., 2019; Hwang Gu et al., 2013). More research on ex-Gaussian parameters analysis can be found in Osmon et al. (2018); Dias (2014).

## Appendix C: Generalized Linear Model

The findings from Feldmann-Wüstefeld et al. (2015) indicate that participants who perceived color as response-relevant in a learning task show larger distractor interference (calculated as the RT increase in distractor-present compared to distractor-absent trials) than participants in the shape group. This observed effect is further reflected in our presented model, wherein the distinction in weighting between the history and color maps contributes to capturing this phenomenon (see page 20 and Fig. 9).

Another influence on attention guidance reflected in our model is the intertrial effect. The results of the model comparison underscore an enhanced fit to the data when intertrial effect is considered (see Fig. 6).

To corroborate our findings, we conducted a generalized linear model analysis on reaction times (RTs). This model explores how the conditions ($Cond=\left\{ trial~type, distractor\right.$ $\left. present, intertrial~effect \right\}$) influence the parameters of the ex-Gaussian RT distribution:

11 $$\begin{aligned} \forall m:\mu [m] = \sum _{i \in Cond}(W_\mu ^i \cdot X^i[m]) + B_\mu \nonumber \\ \sigma [m] = \left[ \sum _{i \in Cond}(W_\sigma ^i \cdot X^i[m]) + B_\sigma \right] _+ \\ \tau [m] = \left[ \sum _{i \in Cond}(W_\tau ^i \cdot X^i[m]) + B_\tau \right] _+ \nonumber \end{aligned}$$

where *W* and *B* are weights and biases of a linear model for the ex-Gaussian distribution parameters $(\mu , \sigma , \tau )$ per each trial *m*. The $X^i$ are indicator variables $\in \{0,1\}$. For trial *m*, $X^i[m]=1$ if trial *m* fulfils condition *i*, and $X^i[m]=0$ otherwise. Condition “intertrial effect” is specifically a within-task intertrial effect and applies if both trial *m* and trial $m-1$ belong to the same task type, either learning or search. $[z]_+ = \frac{1}{20}\log (1+\exp (20\cdot z))$ is a softplus function that constrains $\sigma$ and $\tau$ to be positive.

The weights (*W*) and biases (*B*) are determined by minimizing the loss function:

12 $$\begin{aligned} Loss= & {} - \sum _{m=1}^M \log \bigg (ExG \big ( RT[m]\,|\,\mu [m], \sigma [m], \tau [m] \big ) \bigg )\nonumber \\{} & {} - \log p(\theta ) \end{aligned}$$

where ExG is the ex-Gaussian density function (see Eq. 10) and $p(\theta )$ contains the prior distributions over the model parameters ($W_\mu$, $W_\sigma$, $W_\tau$, $B_\mu$, $B_\sigma$ and $B_\tau$). These priors are:

13 $$\begin{aligned}&W_{\mu |\sigma |\tau }\sim \mathcal {N}(0.0,10.0) \nonumber \\&B_\mu \sim \mathcal {N}(600.0,100.0) \nonumber \\&B_{\sigma } \sim \mathcal {N}(75.0,4.0) \\&B_\tau \sim \mathcal {N}(200.0,20.0) \nonumber \end{aligned}$$

We compared five Generalized Linear Model (GLMs) with the conditions combinations shown in Fig. 14 by computing a Laplace approximation (LAP) to the logarithm of the model evidence, summed across all participants. Figure 14 shows the LAP relative to the baseline model, which does not include any condition information. The results indicate that all the aforementioned conditions positively contribute to predicting participants’ reaction times. The approximate log of Bayes factor (Kass & Raftery, 1995) for the comparison of GLM1 (the linear model with all conditions) and GLM3 (when intertrial effect is excluded) was $BF_{\log _{e}}(GLM1/GLM3)=830.3$, indicating “decisive evidence” in favor of GLM1 and the role of intertrial effect. The log of Bayes factor for GLM1 vs. GLM2 (where distractor information was excluded) was $BF_{\log _{e}}(GLM1/GLM2)=254.5$, also providing decisive evidence in favor of distractor effects on RTs.

When comparing the weights ($W_\mu$, $W_\sigma$ and $W_\tau$) for each condition, group differences become evident, see Fig. 15. We plotted the condition-dependent weights (*W*) that determine ex-Gaussian distribution’s means ($\mu + \tau$). While there is hardly a group effect on the “trial type” differences between the RT means, the “distractor” clearly has a stronger effect on the color group than on the shape group. This difference is about $9.3 \pm 2.8$, see Fig. 15. “Intertrial effect” is strong on both groups (color group: $-30.5 \pm 4.1$, shape group: $-24.2 \pm 3.2$). Furthermore is more effective in reducing the mean for color group members than for shape group members ($-7.4 \pm 5.2$).

## Footnotes

1 A preliminary version of this modeling study has been presented at COGSCI 2021 https://cognitivesciencesociety.org/cogsci-2021/.
